# Regarding the article “Entrustable Professional Activities (EPAs) for the residency program in vascular surgery”

**DOI:** 10.1590/1677-5449.202400072

**Published:** 2024-09-27

**Authors:** Jorge Luis Carrera Martínez

**Affiliations:** 1 Hospital Provincial General Docente “Dr. Antonio Luaces Iraola”, Ciego de Ávila, Cuba.

Dear editor,

The publication of the article “Entrustable Professional Activities (EPAs) for the residency program in vascular surgery” addresses an interesting and relevant issue by expressing the importance of medical education in graduate studies, particularly in vascular surgery residency, as the process of preparing for specialized medical care.^1^

In Cuba, as well as in Brazil, medical education has experienced changes towards its improvement in accordance with international educational models and contemporary scientific challenges, adapted to the Cuban reality. The search for medical education as an open and flexible process of building professional competencies, as well as professional development activities that enable to ensure an effective professional performance,^2^ are translated as safety and efficacy milestones.

Specifically in the training of Cuban resident physicians in Angiology and Vascular Surgery, safety is established in a curriculum that is structured and organized both in teaching and learning. It provides for teaching, attentional, research, and academic management integration with community and hospital units and services, so as to qualitatively improve teaching-attentional activities using the principle of “learning by doing,” translated into Entrustable Professional Activities.

Furthermore, in these Entrustable Professional Activities, Cuban Angiology and Vascular Surgery residents are linked to a hospital service, in order to comply with the identified and standardized competencies to be achieved, taking on responsibilities gradually, under permanent supervision, while acquiring the work habits of the specialty.^3^ Such competencies include those required to perform diagnosis, clinical and surgical treatment, education, research, and care provision to patients with diseases of vascular origin, which is translated into efficacy.

Dear editor, certified vascular physicians are required to search for permanent professional improvement,^4^ so as to ensure incorporation and development of competencies and skills such as novel techniques for the diagnosis and treatment of peripheral vascular diseases. Moreover, professional improvement enhances personal and group development, thus promoting a better quality medical care.
