# Supplementary material for: Regarding the article “Entrustable Professional Activities (EPAs) for the residency program in vascular surgery”
Source: J Vasc Bras. 2024 Sep 27;23:e20240007. doi: 10.1590/1677-5449.202400072 (PMC11573362; doi:10.1590/1677-5449.202400072)
Supplement: Translation

# Translation: A propósito del artículo “Entrustable Professional Activities (EPAs) for the residency program in vascular surgery”

**DOI:** 10.1590/1677-5449.202400073

**Authors:** Jorge Luis Carrera Martínez

**Affiliations:** 1 Hospital Provincial General Docente “Dr. Antonio Luaces Iraola”, Ciego de Ávila, Cuba.

Estimado editor,

Resulta interesante y significativa la publicación del artículo “Entrustable Professional Activities (EPAs) for the residency program in vascular surgery”, al expresar la importancia que se le concede a la educación médica en el posgrado, en particular a la residencia en cirugía vascular, como proceso de preparación para la atención médica especializada^1^ .

Al igual que en Brasil, en Cuba la educación médica ha experimentado transformaciones en aras de su perfeccionamiento, en correspondencia con los modelos educativos internacionales y los retos científicos contemporáneos, atemperados a la realidad cubana. La búsqueda de una educación médica como proceso abierto y flexible de construcción de competencias profesionales, así como las actividades de superación profesional que posibilitan garantizar un desempeño profesional efectivo^2^ , se traducen como los pilares de seguridad y eficacia.

Específicamente en la formación del médico residente de Angiología y Cirugía Vascular cubano, la seguridad se establece en el currículo, estructurado y organizado, tanto la enseñanza como el aprendizaje. En este, se establece la integración docente, atencional, investigativa y de gestión académica con las unidades y servicios de salud comunitarios y hospitalarios, para incrementar cualitativamente las actividades docente-atencionales con el “aprender haciendo”, lo que se traduce como Actividades Profesionales Confiables.

Asimismo, en esas Actividades Profesionales Confiables, el residente de Angiología y Cirugía Vascular cubano es insertado en un servicio hospitalario, con vistas al cumplimiento de las competencias identificadas y normalizadas a alcanzar, con responsabilidades graduales, bajo supervisión permanente; adquiriendo al mismo tiempo los hábitos del trabajo de la especialidad^3^ . Dichas competencias son las necesarias para realizar el diagnóstico, tratamiento clínico y quirúrgico, educación, investigación y prestación de atención a pacientes con enfermedades de origen vascular, lo que se traduce como eficacia.

Señor editor, el médico vascular certificado necesita superación profesional permanente^4^ , para garantizar la incorporación y desarrollo de competencias y habilidades como técnicas novedosas para el diagnóstico y tratamiento de las enfermedades vasculares periféricas. Además, la superación profesional potencia así su desarrollo personal y grupal lo que permite una mejor calidad en la atención médica.
