# Supplementary material for: Regarding the article “Entrustable Professional Activities (EPAs) for the residency program in vascular surgery”
Source: J Vasc Bras. 2024 Sep 27;23:e20240007. doi: 10.1590/1677-5449.202400072 (PMC11573362; doi:10.1590/1677-5449.202400072)
Supplement: Related texts

# Reply: RESPONSE LETTER

**Authors:** Marcos Aurelio Perciano Borges, Ricardo André Viana Barros, Alcides José Araújo Ribeiro, Vanessa Dalva Guimarães Campos

**Affiliations:** 1 Hospital de Base do Distrito Federal – HBDF, Brasília, DF, Brasil.; 2 Escola Superior de Ciências da Saúde – ESCS, Brasília, DF, Brasil.

Dear editor,

We believe that Entrustable Professional Activities (EPAs)^1^, represent an advance with regard to competencies, especially in terms of residents’ evaluation, making it possible for preceptors to provide more objective assessments, because these activities enable to characterize the level of autonomy and supervision of each resident, considering their subjectivity and academic background. Competency-based assessment and that based on EPAs are not exclusionary, but the latter provides greater autonomy to residents; greater objectivity to preceptors’ assessments, and greater safety to patients treated by the team.

The pertinent and interesting comment regarding the article shows that such teaching practice has already been fully developed and implement in different countries, including in Latin America, with the purpose of improving the quality of vascular surgeons’ training, with adaptations to the reality of each society.

## References

[1] Borges MAP, Barros RAV, Ribeiro AJA, Campos VDG (2023). Entrustable Professional Activities (EPAs) for the residency program in vascular surgery. J Vasc Bras.

# Reply: RESPUESTA

**Authors:** Marcos Aurelio Perciano Borges, Ricardo André Viana Barros, Alcides José Araújo Ribeiro, Vanessa Dalva Guimarães Campos

**Affiliations:** 1 Hospital de Base do Distrito Federal – HBDF, Brasília, DF, Brasil.; 2 Escola Superior de Ciências da Saúde – ESCS, Brasília, DF, Brasil.

Estimado editor,

Creemos que las Actividades Profesionales Confiables (APCs)^1^ constituyen un avance con relación a las competencias, principalmente en lo que atañe a la evaluación del médico residente, posibilitando al preceptor mayor objetividad en las evaluaciones, pues permiten la caracterización del nivel de autonomía y supervisión de cada residente, considerando su subjetividad y trayectoria académica. La avaluación en competencias y aquella basada en las APCs no son excluyentes, pero la última establece mayor autonomía al residente, objetividad en las evaluaciones al preceptor, y seguridad a los pacientes atendidos por el equipo.

El oportuno e interesante comentario sobre el artículo demuestra que tal práctica pedagógica ya se encuentra en pleno desarrollo e implementación en diferentes países, incluso en América Latina, con el fin mejorar la calidad de la formación del cirujano vascular, adecuándose a la realidad de cada sociedad.
